# How structural changes in online gambling are shaping the contemporary experiences and behaviours of online gamblers: an interview study

**DOI:** 10.1186/s12889-022-14019-6

**Published:** 2022-08-26

**Authors:** Nerilee Hing, Michele Smith, Matthew Rockloff, Hannah Thorne, Alex M. T. Russell, Nicki A. Dowling, Helen Breen

**Affiliations:** 1 Experimental Gambling Research Laboratory, CQUniversity, University Drive, Bundaberg, QLD 4670 Australia; 2 Experimental Gambling Research Laboratory, CQUniversity, 44 Greenhill Rd, Wayville, South Australia 5000 Australia; 3 Experimental Gambling Research Laboratory, CQUniversity, 400 Kent St, Sydney, NSW 2000 Australia; 4 School of Psychology, Deakin University, 75 Pigdons Rd, Waurn Ponds, Geelong, VIC 3216 Australia; 5 Melbourne Graduate School of Education, University of Melbourne, 234 Queensberry Street, Parkville, VIC 3053 Australia; 6 Southern Cross University, Military Rd, East Lismore, NSW 2480 Australia

**Keywords:** Internet gambling, Wagering, Gambling harm, Gambling disorder, Problem gambling, Access, Inducements, Complex bets

## Abstract

**Background:**

Over the last decade, the provision of online gambling has intensified with increased access, enhanced betting markets, a broader product range, and prolific marketing. However, little research has explored how this intensification is influencing contemporary gambling experiences. This study focused on two research questions: 1) What changes in online gambling have online gamblers observed over the past decade? 2) How have these changes influenced the online gambling experiences and behaviours reported by treatment-seeking and non-treatment-seeking gamblers?

**Methods:**

Two samples of Australian adults were interviewed: 1) 19 people who had been gambling online for at least a decade and with no history of treatment-seeking for online gambling, and 2) 10 people who had recently sought professional help for an online gambling problem. Telephone interviews were semi-structured, with questions that encouraged participants to consider how their online gambling, including any harmful gambling, had been influenced by changes in operator practices and online gambling environments. Data were analysed using thematic analysis.

**Results:**

Both treatment- and non-treatment-seekers noted the increased speed and ease of online gambling, which now enables instant access from anywhere at any time and increased their gambling opportunities. Both groups highlighted the continued proliferation of advertising and inducements for online gambling, particularly during televised sports and racing events, in social media, and through targeted push marketing. Many treatment- and non-treatment-seekers were aware of the vast range of recently introduced bet types, particularly multi-bets. Treatment-seekers disproportionately reported negative effects from these changes, and described how and why they fostered their increased gambling, impulsive gambling, persistence and loss-chasing. They reported limited uptake and effectiveness of current harm minimisation tools.

**Conclusions:**

Counter to stated policy and practice objectives to minimise gambling harm, industry changes that have made online gambling easier, faster, and more heavily incentivised, and increased the array of complex bets with poorer odds, unduly affect addicted and harmed individuals – who are also the most profitable customers. Further consideration is needed to ensure gambling policy, industry practices and public health measures more effectively reduce gambling harm in contemporary settings. Inducements and the poor pricing of complex bets such as multi-bets, and their outsized attraction to players with problems, should be a key focus.

## Background

Online gambling first became available in the 1990s and has since rapidly expanded in scope and availability. Globally, millions of adults now gamble using internet-connected devices, including smartphones, computers and tablets. Past-year prevalence of online gambling appears to be particularly high in Nordic countries, reaching 37% in Norway [1] and 36% in Finland [2]. By comparison, rates are substantially lower in the United Kingdom (21%) [3], Australia (17.5%) [4], and Canada (6.4%) [5]. These different rates reflect jurisdictional variations in the introduction, legality and practicalities around provision of online gambling products. Nonetheless, online gambling has continued to increase over time in countries where it has been legalised [6], fundamentally changing the way that many gambling products are provided and consumed.

Studies have identified several features that distinguish online gambling from land-based gambling that may facilitate gambling participation, problems, and harm (e.g., [7–10]). These include instant 24/7 access from any location; its immersive, private, and solitary nature; use of digital money; the speed of betting transactions; and receiving gambling advertising directly on a gambling device. However, features of online gambling have not remained static, with recent developments characterised as “complex, dynamic and fast moving” ([11], p.1). The provision of online gambling has intensified with increased access, enhanced betting markets, a broader product range, and prolific marketing; all changes that may influence the experience of contemporary online gamblers. At the same time, harm minimisation tools that aim to help people to self-regulate their online gambling have increased.

## The intensification of online gambling

### Increased access

Since the inception of online gambling, internet access has increased dramatically, allowing more people to gamble online [12]. Smartphones now enable immediate and location-independent access to online gambling, allowing gambling to be integrated into everyday activities at home or work, while commuting, in social settings, and when watching betting events [13–16]. Faster internet speeds and streamlined financial transactions on gambling websites and apps have also accelerated the betting process [17, 18].

### Enhanced betting markets

A major change over the last decade has been the continued “industrialisation” of online gambling, spawning an ecosystem characterised by multinational gambling operators, mass-media supported sports and races, digitalisation of betting products, and increased gambling sponsorship and advertising [19–22]. This corporatisation of the industry has manifested in several changes, as operators jostle to succeed in an industry with strong competition, limited scope for product differentiation, and low switching costs for customers [23, 24]. Competitive strategies include the provision of varied online gambling opportunities, product innovations and extensive marketing.

### A broader product range

Operators now provide more online betting options than ever before. The volume of “bettable” sports, racing and esports events has expanded globally, with increased broadcast coverage on television, streaming and mobile platforms [19, 25]. Combined with 24/7 access, customers can now watch and bet on a near-unlimited array of domestic and international events across time zones [20]. Online casinos provide an extensive range of products and enable simultaneous gambling on several games [26]. New gambling forms have emerged, including betting on daily fantasy sports, esports and an increased array of novelty events, although their uptake has been relatively modest [4, 27, 28]. Skins and cryptocurrency provide expanded payment options and enable anonymous expenditure [29, 30].

Consumers have widely adopted extensive innovations in bet types. Bets can now be placed before and after match commencement and on numerous in-match contingencies, such as half-time scores, increasing each event’s betting markets [31, 32]. In-play betting elevates the risk of gambling harm since it enables bettors to place more bets per event, engage in high-speed continuous betting, and persist and extend online betting sessions [18, 33]. Research indicates higher rates of harmful gambling amongst in-play bettors [34–36], including those who bet on micro events, an accelerated form of in-play betting requiring rapid decision-making [37, 38]. Novel betting products also enable changes to betting decisions during play. Using cash-out options, betting becomes an increasingly continuous activity with heightened potential for loss of control, irrational decisions, impulsive gambling, increased emotional involvement, and illusions of control [39–41]. Moreover, cashing out is associated with increased likelihood of gambling problems [36, 42]. Other innovated bet types, such as accumulators, multi-bets and complex bets, may have similar effects because they typically have less favourable odds, plus other structural characteristics likely to increase susceptibility to gambling harm [32, 41, 42].

### Prolific marketing

Increased industry competition has spawned the intensification of advertising for online gambling. This advertising is extensive in social media, online channels, and direct messaging via emails, texts and push notifications [43–45]. Gambling operators have continued to increase their social media presence, use of social influencers (e.g., affiliate marketers), and advertisements on streaming platforms and gaming apps [30, 46]. Television advertising remains extensive, particularly during sports and racing events [43, 47–49]. Online gambling operators also gain extensive brand exposure as sponsors of sports and races [24]. Overall, gambling advertising is highly targeted, concentrated in sports and social media, and focuses on promoting brand awareness, complex bets with long odds, and financial inducements to bet [44, 50]. Financial inducements have become a mainstay. They incentivise betting through offering “something for nothing” such as matching deposits and bonus bets, or “reduced risk” such as refunds and cash-out options [19, 32, 51, 52]. Embedded in digital media, consumers can click on a link in the promotion to immediately place the bet [45, 53].

### Harm minimisation tools in online gambling

The intensification of online gambling has been accompanied by the introduction of several consumer protection tools. For example, the Australian Government is implementing the National Consumer Protection Framework for Online Wagering. Measures include a voluntary opt-out pre-commitment scheme for setting deposit limits on betting accounts. Additional tools yet to be introduced include player activity statements, consistent safe gambling messaging, and a national self-exclusion register. Most licensed operators already provide options for player activity statements, limit-setting and self-exclusion. Only a minority of customers use these tools [4]. Lower-risk gamblers are resistant because they already feel in control of their gambling [54–56], while higher-risk gamblers may not want to limit their gambling [57] or find limits and self-exclusion easy to circumvent by opening additional accounts [58]. Nonetheless, customers who use harm minimisation tools tend to find them useful [55, 56, 59].

Despite the rapidly changing industry dynamics discussed above, there is limited research on how the greater scope and variety in the provision of online gambling is influencing contemporary gambling experiences specifically for online gamblers as opposed to gamblers in general. A recent review noted the need for qualitative studies to better understand emerging technologies and new trends in gambling [25]. The current study helps to redress this need, focusing on Australia, where online gambling is now the fastest growing form of gambling, especially on sports, races and lotteries which can be legally provided to residents [4].

## Methods

### Study aims, design and setting

The study aimed to better understand emerging technologies and new trends in gambling through a qualitative interview study based on the lived experiences of online gamblers in Australia. It focuses on two research questions:

1. What key changes in online gambling have online gamblers observed over the past decade?
2. How have these changes influenced the online gambling experiences and behaviours reported by treatment-seeking and non-treatment-seeking gamblers?

Understanding how these recent changes may have influenced gambling and related harm for online gamblers is important to inform contemporary policy and harm minimisation measures. While numerous studies have provided cross-sectional quantitative data on online gambling behaviour (e.g., [1, 2, 4, 60]), limited research has drawn on gamblers’ lived experiences to understand how recent changes in online gambling influence their gambling choices.

#### Recruitment and samples

The study recruited two samples of interviewees from a database of participants in the researchers’ prior gambling studies (references blinded for review) who had agreed to be invited into further research. Inclusion criteria were aged 18 years or over; living in Australia; and either: (a) reporting gambling online in our 2012 survey on online gambling, and reporting gambling online at least fortnightly in our 2020 survey on online gambling, and with no history of treatment-seeking for online gambling (non-treatment-seekers); or (b) having sought treatment for problems with online gambling in the last three years (recent treatment-seekers). In this context, treatment-seeking meant they had sought professional help for problems relating to their online gambling, from a face-to-face service, telephone, or online service. Recruiting these two samples enabled the exploration of perceived changes in online gambling over the past decade, as well as how these changes may have differentially impacted on those who had, versus those who had not, sought professional help for their online gambling.

Potential participants across a range of ages, genders, and locations were invited via email to participate in an interview. To avoid oversampling, email invitations were sent in batches of 20 to potential participants in the non-treatment-seeker group. To recruit the target of 20 participants, 102 people were emailed, yielding a response rate of 19.6%. Email invitations were sent in batches of 20 (and then 50) to potential participants in the treatment-seeker group. To recruit a target of 10 participants, 452 individuals were emailed, yielding a response rate of 2.2%. These sample sizes were prearranged with the funding agency and based on pragmatic decisions about what was achievable within the project timelines and budget. This sampling decision also recognised the inherent greater difficulty of recruiting participants who had sought professional help for problems relating to their online gambling, which is reflected in the lower response rate for this cohort. As noted by Braun and Clarke [61], determining sample size relies on a combination of interpretative, situated, and pragmatic judgment about how many participants are needed to enable a rich analysis of patterns related to the research topic, and the number required for data saturation cannot be known in advance [62]. Ideally, sample size should be adjusted during data collection to reach saturation. This was not possible as in the current study the funding agency required definitive sample sizes in advance of the research. Therefore, data saturation may not have been achieved with these prearranged sample sizes.

#### Procedure

Individuals who expressed interest in participating were emailed a link to an information sheet and consent form, which included contact details for help services. Those who consented were then phoned to confirm eligibility and arrange an interview time. One researcher conducted telephone interviews with non-treatment-seekers, and one provisionally registered psychologist conducted telephone interviews with treatment-seekers. The interviews were semi-structured, with questions and prompts to encourage participants to consider how their online gambling, including any harmful gambling, had been influenced by changes in operator practices such as advertising, inducements, gambling products and financial transactions; and changes in online gambling environments such as online and mobile access. Interviews lasted for between 45 and 60 min and were professionally transcribed. Participants received a $50 shopping voucher.

#### Participants

Thirty participants from five Australian states were interviewed. This included 20 non-treatment seekers, aged between 32 and 87 years (*M* = 55.9 years), but one interviewee’s data was subsequently excluded from analysis after disclosing prior treatment-seeking for online gambling many years earlier. Of the remaining 19 participants, 18 were male, and they mainly gambled on sports and races using a smartphone. Nine male and one female treatment-seeking gamblers, aged between 21 and 68 years (*M* = 41.8 years) participated. Seven gambled mainly on sports and races, two on online slots, and one on online poker, mostly using a smartphone. Tables 1 and 2 summarise the key demographic characteristics and gambling behaviours of participants.

**Table 1:** Key characteristics of non-treatment-seeking (NTS) online gamblers

ID	Age	Sex	State	Main online gambling form	Main devices used	Online gambling frequency per week	Online gambling AUD$ per week
NTS1	36	F	SA	Sports betting, some race betting	Smartphone	2–3 times a week	$100–150
NTS2	41	M	SA	Sports betting, spread betting, arbitrage	Smartphone	Weekends	$50–100
NTS3	50	M	NSW	Race betting, some sports betting	Smartphone, computer	Weekly	$300–400
NTS4	56	M	SA	Race betting, some sports betting,	Smartphone	Weekends	$10–100
NTS5	87	M	WA	Sports betting, novelty events	Computer	2–3 times a week	$5 minimum each bet, then build upwards
NTS6	32	M	SA	Sports betting, some race betting	Smartphone	Weekly	$200–250
NTS8	47	M	SA	Sports betting, some race betting, informal punters club	Smartphone, laptop	Weekends	$100–150
NTS9	52	M	QLD	Race betting, some sports betting	Computer, smartphone	6 days a week	$2000 bet on each race, laid off by spreads betting, possibly $12,000 minimum weekly t/o
NTS10	56	M	QLD	Race betting, sports betting, informal punters club	Computer	2 days a week	$25
NTS11	65	M	QLD	Race betting	Computer	2 days a week	$2–10 each bet
NTS12	67	M	NSW	Race betting, sports betting	Smartphone	2–3 days a week	$10–100 each bet
NTS13	69	M	SA	Sports betting, race betting	Laptop	Daily	$2000 bet on sports events laid off by spreads betting
NTS 14	73	M	VIC	Sports betting, novelty events, previously horse racing	Computer	Weekends	$100–200
NTS 15	36	M	NSW	Race betting	Smartphone, computer, tablet	Weekends	$150
NTS 16	57	M	VIC	Race betting	Computer	4 days a week	1–8 bets per race × 8 races per day, Liability < $100 per race
NTS 17	68	M	NSW	Race betting, sports betting, novelty events, informal punters club	Smartphone	Weekends	$20–25 per bet
NTS 18	47	M	VIC	Race betting, sports betting, novelty events, informal punters club	Smartphone, tablet	2 days a week	$2000 per race. Less with sports bets, ~ $1000 bets on football
NTS 19	53	M	WA	Sports betting, some race betting	Smartphone	Weekly	$10–20 each bet
NTS 20	83	M	WA	Race betting, some sports betting	Computer	Daily	N/A

*NSW* New South Wales, *VIC* Victoria, *QLD* Queensland, *SA* South Australia, *WA* Western Australia

**Table 2:** Key characteristics of treatment-seeking (TS) online gamblers

ID	Age	Sex	State	Main online gambling form	Main devices	Online gambling frequency per week	Online gambling AUD$ per week
TS1	21	M	SA	Race betting, previously sports betting, novelty bets	Laptop, smartphone	2–3 times	$100-$150
TS2	21	M	QLD	Race betting, sports betting	Smartphone	5 days	$200-$250
TS3	63	F	QLD	Online slots	Smartphone	Every day, now stopped	Unsure, but caused poverty & debt
TS4	38	M	VIC	Sports betting, race betting	Smartphone, tablet	5 days	$400-$500
TS5	41	M	WA	Sports betting	Unclear	~ 10 bets	$150
TS6	49	M	VIC	Race betting	Smartphone, computer	1 now, previously much more	Now $35-$45, previously ‘a heck of a lot more’
TS7	32	M	NSW	Online slots	Smartphone	1–2 days, could play all night	$300, previously $750
TS8	36	M	VIC	Online poker	Laptop, tablet	6–10 h	$100-$150
TS9	68	M	NSW	Race betting, sports betting	Smartphone	Every day	Turnover $7,000-$10,000
TS10	49	M	VIC	Race betting	Smartphone, computer	Nearly every day	$400-$2,000

*NSW* New South Wales, *VIC* Victoria, *QLD* Queensland, *SA* South Australia, *WA* Western Australia

#### Analysis

Data were analysed using Braun and Clarke’s protocols for thematic analysis [63]. After data familiarisation through multiple readings of the interview transcripts, the analyst generated initial codes by systematically working through each transcript and collating the codes into potential themes and sub-themes using an iterative process of review and refinement. To enhance trustworthiness, the analysis was checked by the interviewers and a second researcher, with further refinements made to ensure it faithfully captured important aspects of the lived experience reported by participants. Participants’ quotes from non-treatment-seeking (NTS) and treatment-seeking (TS) subgroups are used to highlight types of content that informed the construction of the themes in the results that follow.

#### Ethics

The study was approved by the Human Research Ethics Committee at CQUniversity (reference: 22230).

## Results

The analysis identified several themes and subthemes relating to perceived changes in online gambling over the past decade, and how these changes were perceived to influence the online gambling behaviour of participants (Table 3).

**Table 3:** Themes and sub-themes from interviews with treatment-seeking and non-treatment-seeking online gamblers

Theme 1. Changes in the accessibility, ease and speed of online gambling ∙ Increased speed and ease ∙ Increased accessibility ∙ Faster financial transactions ∙ Reported effects of increased ease, speed and access to online gambling
Theme 2. Changes in the advertising of online gambling ∙ Increased advertising ∙ Increased social media advertising and push marketing ∙ Reported effects of advertising on online gambling
Theme 3. Changes in inducements for online gambling ∙ Amount and types of inducements ∙ Reported effects of inducements on online betting
Theme 4. Changes in betting products ∙ New bet types used ∙ Reported effects of new bet types on online gambling ∙ Newer forms of online gambling ∙ Reported effects of newer forms on online gambling
Theme 5. Use of harm minimisation tools ∙ Player activity statements ∙ Deposit limits ∙ Self-exclusion ∙ Perceived adequacy of harm minimisation tools

### Theme 1. Changes in the accessibility, ease and speed of online gambling

#### Increased accessibility

Participants highlighted how smartphones have increased access to online gambling anywhere and anytime, whereas “10 years ago, I just had to come home and do it on the laptop…time and place that you can place a bet [have increased]” (NTS8). Most non-treatment-seekers preferred to gamble using a smartphone at home, describing the ease, comfort, convenience, anonymity, and quieter environment compared to a sports event or venue. Some said this gave them more time to research betting markets and make informed decisions. Others felt it enhanced their discipline and control over gambling compared with being out in venues drinking with friends, placing larger bets more often, and chasing losses.

Treatment-seekers also preferred to gamble from home, with most using a smartphone. Two did not drive due to medical conditions and two others sought to avoid unpleasant, noisy, or intoxicated people and cigarette smoke in venues. Four treatment-seekers preferred the privacy of online betting to avoid feeling stigmatised. One who described “significant impacts” from his online gambling, including depression and losing his family’s trust, explained:

It’s very private and that’s a good feeling. No one’s watching you, no one’s judging you…Because of my history, I’ve still got this paranoia…I don’t want people to see me. (TS6)

All treatment-seekers discussed how easy, quick and convenient it was to gamble from home, without the effort of visiting a venue. “You’ve got to get up…get changed…drive down there…Where you can just sit in your pyjamas…[and] bet at 6am on your couch” (TS4). Another treatment-seeker who had “ruined a couple of interpersonal relationships” due to his gambling and associated lying noted: “You can just be in the comfort of your own home. Throwing your money in the bin there instead” (TS7). Access to international events provided around-the-clock betting opportunities on races and sports. “Now it’s like 24 h. Back when I was younger, it stopped and there was no international racing” (TS10). “And sport is on every day of the week, 24/7 virtually” (TS4).

#### Increased speed and ease

Eleven non-treatment-seekers commented that increased internet speeds enabled instantaneous gambling, including in-play betting, easy use of gambling websites and apps, and access to the latest betting information. Smartphones enhanced this instant availability: “You’ve got so much information now…on the app on the phone, you can get the form… replays…podcasts” (NTS17). In contrast, “years ago…you had to wait for the results to come in…now…everything…is instantaneous” (NTS1).

Treatment-seekers noted how quickly and easily they could act on betting information and start betting: “I can go from turning my phone on to having a bet on in the space of 20 s…I don’t need to be getting anywhere or making a phone call” (TS2).

#### Faster financial transactions

Non-treatment-seekers reported that faster methods to deposit and withdraw money facilitated betting transactions and made online gambling more attractive. Several treatment-seekers reported beneficial changes, such as recent shorter delays in withdrawing funds, which reduced the temptation to gamble winnings. Treatment-seekers also reported downsides, such as placing bets with a single button press, making it easy to spend large amounts. One treatment-seeker reported a “massive impact” because deposits that previously could not be accessed until the following day were now instantly available for betting (TS10). This change removed the delay that had helped him control his betting by preventing him from immediately chasing losses. He described the “trap” which enabled easy transfers from bank accounts to betting accounts to facilitate continued betting and loss-chasing. Another treatment-seeker reported that withdrawals could be cancelled within an eight-hour window, which facilitated chasing losses (TS2).

While financial transactions had become faster, treatment-seekers reported that some operators required betting account verification to withdraw money, but not to open an account and make deposits. Account verification might take several days, in which time any winnings might be gambled away:

So easy to deposit money…In five seconds, bang…then some of them make it very hard to withdraw…it can be 24 hours to verify your account…by that time, your money has been spent already. (TS6)

#### Reported effects of increased ease, speed and access to online gambling

Non-treatment-seekers observed that the increased ease and speed of online gambling increased its potential for harm, and three reported periods of impaired control. Reduced cooling-off periods between bets increased the likelihood of chasing losses:

If you’ve got to ring up…you’ve got a bit more of a cooling-off period than if you’re sort of doubling down. If you can put the punts online…it speeds things up, and it creates that possibility” (NTS19).

Another non-treatment-seeker described “going on tilt” when his online betting became reckless and uncontrolled, with escalating losses resulting in emotional frustration and abandonment of planned betting strategies (NTS2). Most non-treatment-seekers, however, did not report any harmful effects of changed access to online gambling, explaining they prioritised their family’s welfare, knew when to stop, or set limits on their betting.

In contrast, treatment-seekers reported that more convenient and easier access to online gambling had increased their gambling frequency and expenditure: “If I didn’t have access to online gambling, my gambling would be reduced by 80%” (TS2). Several treatment-seekers discussed how 24/7 access to online gambling facilitated betting on international events and removed constraints such as venue closing times. One participant who had experienced “deep financial problems” from playing online slots (pokies) explained, “When you go to a regular club, they close… With online pokies, it was…24 h a day, seven days a week…That certainly contributed to me doing it more” (TS3).

Treatment-seekers acknowledged that the privacy afforded by online gambling, particularly on a smartphone, made it easier to hide from family: “You can gamble online more sneakily…because you can just do it on your phone and you could be saying, ‘I’m just texting a friend’” (TS10). Three treatment-seekers were drawn to the immersive qualities of online gambling because it took their mind off worries. “I could lose myself in it…a totally different world…take me out of myself for a while…I would do [online pokies] every day…hours at a time” (TS3).

### Theme 2. Changes in the advertising of online gambling

#### Increased advertising

All participants noted the proliferation of online gambling advertising across all media platforms, particularly during televised sports events:

It’s in your face. It’s everywhere…the radio station…the shows you watch…Foxtel …newspaper...certain websites and all their bloody ads pop up...Facebook…notifications…on TV…on your computer…a footy match…posters around the stadium…I just don’t reckon there’s a day where [sic] you don’t see something. (TS6)

Treatment-seekers who bet on sports or races further increased their exposure to this advertising by watching programs and networks devoted to sports and racing. “I see it everywhere because I’ve got Skytell on a lot, and TVN” (TS10).

#### Increased social media advertising and push marketing

Participants frequently received targeted social media and push marketing messages for online gambling including emails, notifications, text messages and phone calls. One non-treatment-seeker thought the huge quantity of social media advertising began a few years ago when gambling advertising was restricted during televised sporting events. He described being assigned an account manager “who bugs you and sends you text messages and calls…a phone call every now and then…a text from him pretty much every Friday night…emails” (NTS6).

Treatment-seekers also noted the high frequency of gambling advertising on online and social media platforms: “What I follow is gambling-related, so I see it on Twitter. Even my Facebook page. I see it everywhere” (TS10); “Facebook a lot. I see a little bit on Instagram as well. Snapchat… A little bit on YouTube” (TS2).

#### Reported effects of advertising on online gambling

Some non-treatment-seekers found the advertising irritating, persistent and offensive, and had therefore blocked or disregarded it: “they are trying to be greedy and trying to get you in” (NTS1). However, one said it enticed him to bet, while another indicated that he would normally investigate the advertised offer. A few treatment-seekers said they were not influenced by online gambling advertising because they ignored it or no longer used social media. Others, however, reported that advertising had enticed them to sign up to new betting websites, even after self-excluding from other sites:

I certainly signed up to websites 100% based on seeing new ones pop up [on ads]...I go, ‘Shit, I haven’t joined that one. I’m self-excluded on the others. This is a new one I can join up on. Beauty’. (TS6)

Another treatment-seeker who had experienced considerable financial consequences and subsequently stopped playing online slots, reported that she still received emails from online casinos. She worried that these advertisements still had the power to tempt her to play. Several treatment-seekers reasoned that regulation should limit gambling advertising because it was “overwhelming…especially if you have a problem” (TS8).

### Theme 3. Changes in inducements for online gambling

#### Amount and types of inducements

Sixteen non-treatment-seekers had used inducements, but some no longer received these offers after earlier wins. Non-treatment-seekers reported that inducements remained prolific but had peaked several years ago when industry competition was most intense. Some potentially misleading inducements had been restricted: “bonuses…back in the day, they were unregulated then. They were so rigged it was ridiculous” (NTS2). Non-treatment-seekers observed that inducements now had stricter conditions, such as time limits. Many, however, reported having accounts with multiple operators so they could access the best inducements.

In contrast, treatment-seekers reported that the number and types of inducements had increased rapidly, were advertised by all operators, and included deposit bonuses, bonus bets, bonus credit, price freezes, money-back offers, odds boosts, protest payouts, double your winnings, and free spins and credits on online pokies. They also received inducements through direct marketing:

I’m with so many corporates, one might do it [text me] one week, one might do it the next week…Especially on a Friday…they pump out all the text messages and the promos because most guys will bet on Saturday. (TS10)

#### Reported effects of inducements on online betting

Some non-treatment-seekers acknowledged being drawn in by inducements. One participant noted how enticed he was by bonuses, but recognised the importance of remaining in control:

Deposit $1,000, get a $200 bonus. Why wouldn’t I use it? I’d be mad not to… If you can control your gambling… If you can’t control your gambling, then it’s maybe not a good idea. (NTS9)

Other non-treatment-seekers said they always examined terms and conditions, and researched new operators before signing up: “just suss out exactly what they’re offering…it’s got to be something that really catches my eye for me to think about opening an [additional] account” (NTS6).

Non-treatment-seekers noted carefully assessing the value of inducements before using them. Several researched individual components of combined contingencies to ensure the inducement’s value exceeded what could be obtained without it. Despite this cautious attitude, some non-treatment-seekers reported being attracted by inducements, especially bonus bets because they provided more betting funds.

In contrast, treatment-seeking participants did not report exercising caution or attempting to establish the true value of inducements before taking them up. Instead, they reported being very enticed by inducements: “They’re the lure…Yeah, you jump” (TS6). They reported numerous harmful impacts, including spending more to meet turnover requirements; not reading the conditions and then being ineligible for the bonus or unable to withdraw winnings; placing riskier bets on long shots with money-back offers; or impulsively betting on a promotion before researching bets and then chasing their losses. Some treatment-seekers reported immediately taking up bonus bets, even if it meant spending more than planned:

If I get a phone call saying, ‘Look, we’ll give you up to $250 in bonus bets’, I’ll act straight away…that one is by far the most potent… I could only afford $50 and I ended up spending $250 because they called me. (TS2)

Another treatment-seeker described feeling “a real hypocrite and devious guy” because he continually lied to his family about his gambling, which had also greatly undermined his business’s success. He reported how bonus bets had contributed to his spiraling gambling problem:

Even at nine o’clock this morning because of these bonus bets… I’ve already put my $150 on the first three races already… And if that loses, then I’m in the same old spiral that I’m in every single day. (TS9)

Treatment-seekers also reported shopping around for the best inducements. This increased the number of betting accounts held, time spent on gambling activities, and the number of inducements subsequently received.

### Theme 4. Changes in betting products

#### New bet types used

Multi-bets were popular with some non-treatment-seekers and were the most popular new bet type amongst treatment-seekers. Other exotic bets mentioned by both groups included in-game contingencies (e.g., first score/penalty) and combined contingencies (e.g., team to lead at half-time but lose the match). The prohibition on offering in-play bets online in Australia appears to have deterred their use in this sample. This prohibition also appears to have deterred using cash-out options. Seven non-treatment-seekers now only used a betting exchange after other operators had banned them following their earlier betting success. They were highly critical of corporate bookmakers: “they basically want…losers, people that lose money” (NTS16).

#### Reported effects of new bet types on online gambling

Most participants indicated that recently introduced betting options had greatly increased betting opportunities. One non-treatment-seeker viewed multi-bets as a logical addition after researching and selecting individual bets. While his outlays were modest, multi-bets nonetheless increased his expenditure:

They’ve put out that same race multi…that’s chewed through a few 50 cents for me…it’s in addition… I put my bets down…and then…put a couple of multis…a dollar [each leg]. (NTS11)

Treatment-seekers perceived multi-bets as particularly attractive because they might enable bigger bets following wins from smaller bets in hope of a “life changing…big collect” (TS10). They described how multi-bets increased betting involvement because they were particularly enticing, provided greater choice, and could be selected according to preferred teams and specific contingencies. This could increase the perceived role of skill in betting success: “You’re able to pick who’s going to be the try scorer and when the time of the try is going to be and if they’re going to even convert it” (TS2). Multi-bets could increase emotional involvement in betting:

When I first start…I’m betting $2 a race…and I’m hoping and dreaming that I can get above $50, $60. Then I start betting $10 and then $20, and then I put a $50 on something and then…you’ve lost it all again… That’s pretty much the pattern of my betting. (TS6)

Non-treatment-seekers who placed exotic bets were cautious, ensuring they first understood the odds and conditions. One non-treatment-seeker noted the “overwhelming” choice of exotic bets, and reported sometimes struggling to resist these riskier bets since they were a tempting way to chase losses:

They encourage you to spend money on…a long shot…if you’re starting to lose, you get a bit more desperate so you’re more inclined to take [them] up… I try and be quite disciplined…sometimes it is a struggle…they put out offers that are designed to induce you to take them up. (NTS10)

Only a few treatment-seekers limited their betting to head-to-head bets. Others described the huge range of exotic betting options now available: “There’s no limit to what you can do… It’s basically customisable” (TS2).

Some participants had tried in-play betting but found the telephone system inefficient. Two treatment-seekers, however, reported placing live bets by phone if they thought it might pay off: “if you’re watching a game and you can see momentum changing…that would sway me to ring up and have a bet…maybe put $200 on it” (TS4). Use of cash-out options was mentioned by only one participant, who found it attractive because, “if I take this now, then I’ve got an extra five or six bets’” (NTS10).

Non-treatment-seekers using betting exchanges engaged in arbitrage betting which requires research and significant outlay but low risk by: “backing and laying the same runner or the same competitor in a sporting event with a fairly significant outlay to make a small profit irrespective of the result” (NTS13).

#### Newer forms of online gambling

Most non-treatment-seekers were aware of newer gambling products, including esports betting, daily fantasy sports betting and skin gambling. Only two, however, had engaged with any of them, with the remainder preferring to bet on the activities they understood: “I didn’t want to try some exotic sport that I knew absolutely nothing about because I just didn’t see the value in that” (NTS10). No treatment-seekers reported engaging with these newer products.

#### Reported effects of newer forms on online gambling

No effects of these newer activities were reported, due to this low level of participation.

### Theme 5. Use of harm minimisation tools

Activity statements, deposit limits, self-exclusion, time-out options, and account closure were variously used by participants.

#### Activity statements

Ten non-treatment-seekers regularly used activity statements and compared them to bank statements that inform of deposits and withdrawals. Those who did not use them criticised the clumsy download system to access statements, that the volume of information made them difficult to understand, and that a monthly statement was too old to be useful.

Only two treatment-seekers used activity statements, although one only once after a weekend of big losses. This same participant explained how the statement available on the operator’s site included only his most recent transactions, and that he had to contact the operator directly to obtain a statement covering a longer period. Seeing this full statement prompted him to decrease his gambling somewhat:

Seeing the total was a bit like, “oh geez, got to cut down a bit”. And I did actually, after that. I stopped for like a week…and kind of took stock. And I guess I’ve got it under control a bit more now. (TS2)

#### Deposit limits

Two non-treatment-seekers had set deposit limits as a safeguard, but the remainder reported that they only bet small amounts and could control their gambling: “I haven’t resorted to this type of action…I only gamble what I can afford to lose…if it becomes an issue, yes, I would consider it” (NTS3).

A few treatment-seekers had used deposit limits, and this could curtail some of their impulse betting and reduce financial harm:

I have set limits on how much I can deposit…the impulse betting is a killer. You want to chase a bet but if you can’t get the money into your account, well you just can’t do any more damage. (TS10)

In contrast, others who had set a limit had subsequently increased it when once the minimum time period had elapsed and their self-control waned:

You’ve just got to sit out three weeks and then you can go back to setting whatever limit you want again…I’ve blown that over the years, thinking I’m going to be a good boy and I’m going to set a limit. Then a month down the track, you’re punching in, “No, I want to change it”. (TS6)

#### Self-exclusion

Non-treatment-seekers typically considered that self-exclusion was a helpful tool for other people, but felt that they did not need to use it. Several treatment-seekers had self-excluded from numerous operators, but subsequently opened accounts with other operators. One reported opening new accounts with operators he had excluded from by using his wife’s details. Operators may also try to dissuade self-exclusion by pointing out the difficulties of re-opening the account or that the customer could never re-open an account with them:

I rang them and said, “Look, I’ve got a problem. I need to close my account” and [they said] to re-open it, you’d need a letter by a psychologist or a counsellor or whatever. So basically, it would be costly for you to follow it up and do it…Saying that, I just joined a different one [operator]. (TS5)

Treatment-seekers explained that self-excluding was contingent on reaching the point of wanting to stop gambling and having the willpower to self-exclude. This interviewee described temporarily taking time out instead, but with limited effectiveness:

I wasn’t strong enough to self-exclude so I thought, “I’m just going to have a three-month break from this company”...It could be two days, a week, two weeks later, I’m back into it again because I’m finding another company. So, that doesn’t work, the rest periods. You’ve got to self-exclude permanently. (TS4)

#### Perceived adequacy of harm minimisation tools

Nearly all treatment-seekers considered it unrealistic to expect people with a gambling problem to be able to self-regulate their gambling. They advocated for improved operator practices, including affordability checks, imposed betting limits, timers on betting websites, and a dashboard summarising betting transactions. Some treatment-seekers also thought that operators should proactively monitor for harmful gambling behaviours, intervene to check on the customer’s welfare, and exclude them if necessary.

Treatment-seekers thought that government regulation was needed, because operators would otherwise do little to deter their most profitable customers:

I think the government has a big part in this. They really need to make it tougher…I don’t think the laws are strong enough to stop people, the problem gamblers, definitely not…It has to be with the government…because problem gamblers are the ones that most of these companies make their money from. (TS5)

## Discussion

When online gambling first emerged, researchers identified numerous features that distinguished it from land-based gambling that were likely to elevate its risk of harm (e.g., 7–10]). The current study extends upon that focus to consider how more recent changes in online gambling may be impacting on contemporary gambling behaviour, including harmful gambling. The principal finding is that higher-risk online gamblers, indicated by recent treatment-seeking behaviour, reported the most negative impacts from recent changes that have intensified many aspects of online gambling. These include easier and faster access, continued proliferation of advertisements and inducements, and the expansion of innovated betting products.

### Ease and speed of access

Both treatment- and non-treatment-seekers noted the increased speed and ease of online gambling, which now enables instant access from anywhere at any time [13–15]. Both groups appreciated being able to immediately source betting information and place bets, and the convenience and comfort of gambling from home. Both groups also acknowledged that easy 24/7 access increased their opportunities to gamble. This ability to gamble quickly and easily, with reduced cooling-off periods, had led some non-treatment-seekers to experience episodes of impaired control, but most self-regulated their gambling to within affordable limits. In contrast, treatment-seekers reported increasing their online gambling in response to easy, convenient, and private access to 24/7 betting opportunities without the constraints of needing to visit a venue, venue closing times, or social judgment. Both groups reported that faster financial transactions facilitated betting, but only treatment-seekers discussed associated disadvantages. For them, the ease of transferring funds to betting accounts contributed to impulsive betting and quickly losing large amounts of money, thereby nurturing persistence and loss-chasing. The difficulty of withdrawing funds from betting accounts and being able to cancel withdrawals, also undermined their self-control. These results are consistent with earlier reports by online gamblers that instant 24/7 access to online gambling can facilitate impulsive gambling, long gambling sessions, high expenditure, and loss-chasing, particularly amongst those with higher gambling severity [8, 17, 18, 26]. Higher-risk gamblers tend to be more impulsive [64, 65], while gambling urges, impaired control, persistence, and loss-chasing constitute symptoms of a gambling disorder [66]. Instant access to online gambling allows individuals experiencing these symptoms to immediately act on a gambling urge and persist at gambling, undermining their self-control and exacerbating the harm they are already experiencing.

### Advertisements and inducements

Both groups discussed the continued proliferation of advertising and inducements for online gambling across all media, particularly during televised sports and racing events, in social media, and through targeted push marketing in texts, notifications, and emails. Online gamblers have previously described being inundated by gambling advertisements and being particularly tempted by frequent gambling inducements [8, 67, 68]. Treatment-seekers further increased their exposure to this marketing by watching sports and racing programs, and by following gambling-related content which increased gambling advertising in their social media feeds. Both groups also appeared to be targeted based on their past gambling performance, with successful punters banned from inducements and less successful punters inundated with inducements. Increased exposure may partly explain why treatment-seekers were more persuaded by advertisements, compared to non-treatment seekers, given the dose–response effect between exposure to gambling advertising and gambling behaviour [24, 69, 70]. Further, higher-risk gamblers tend to report greater influence from gambling advertising and inducements [8, 44, 71]. Treatment-seekers described being strongly tempted by this advertising, particularly for wagering inducements, and were more likely than non-treatment-seekers to immediately take up inducements without assessing their value or conditions. This behaviour is consistent with the influence of marketing cues in the development and maintenance of addictive behaviours, where more addicted consumers have lower self-control and stronger urges since their behaviour is more driven by need, heightening the likelihood of harmful consequences [72]. As found in other wagering research [70, 72, 73], treatment-seekers reported that inducements prompted them to spend more than planned, place riskier bets, bet impulsively, chase losses, and reduce the effectiveness of existing self-exclusions by opening new accounts. Thus, minimal constraints on wagering advertisements and inducements, despite substantial community opposition to their proliferation [74, 75], appear to have continued to nurture harmful gambling behaviours amongst higher-risk gamblers.

### Bet types

Many treatment- and non-treatment-seekers were aware of the vast range of recently introduced bet types, with multi-bets the most frequently mentioned. Treatment-seekers discussed how multi-bets elevated their excitement and emotional involvement in betting, their sense of skill in selecting bets, and their hopes of placing larger bets if earlier legs won. Bets on in-game and combined contingencies were also popular. Non-treatment-seekers reported approaching these bets cautiously, including first evaluating their potential value, being aware they were long shots, and recognising the temptation they posed for chasing losses. In contrast, nearly all treatment-seekers had incorporated exotic bets into their betting patterns, including multi-bets, accumulators, and complex bets. These long-odds bets are the least profitable for bettors because of their higher house-edge and because long-term positive returns are unlikely regardless of skill; however, their large potential wins are particularly attractive to higher-risk gamblers [32, 39, 41]. Since a payout requires all contingencies to occur, these bets also increase opportunities for near misses which may motivate further gambling [41]. Overall, our findings support that these newer bet types are particularly attractive to higher-risk gamblers, elevating their likelihood of experiencing further gambling losses and harm.

### Banning of successful bettors

Several interviewees reported that betting operators had banned them, restricted the amount they could bet, or excluded them from promotions and rewards following their earlier betting success. They were highly critical that operators were only interested in more profitable customers who sustained larger losses. While these bettors reported switching to betting exchanges, other Australian research has highlighted that banned gamblers also opt to use illegal offshore sites, which limits consumer protection [76]. Amongst 347 Australian sports or race bettors who had bet with an offshore operator, 13.8% reported “no betting limits or account restrictions” as an advantage of doing so [4]. Thus, banning successful bettors appears to drive some customers to unlicensed wagering operators who may implement few, if any, harm minimisation measures.

### Use of harm minimisation tools

Most interviewees were aware of harm minimisation tools for online gambling, including player activity statements, deposit limits and self-exclusion. However, their uptake and apparent effectiveness were limited. Non-treatment-seekers thought they did not need to use these tools, although some used player activity statements to stay informed about their gambling spend. Treatment-seekers had used a range of tools, but their effectiveness was typically short-lived and undermined by the ease of changing limits and opening new accounts to circumvent self-exclusion. They advocated strongly for regulation requiring operators to proactively conduct affordability and customer welfare checks, monitor for harmful gambling behaviours, and exclude customers if needed. They thought that relying on self-regulatory tools was unrealistic, given their impaired control over gambling.

### Regulatory effectiveness

Some of the study’s findings point to the potential effectiveness of regulations in limiting gambling harm. In-play bets and cash-out options were not widely reported in this sample, most likely because they cannot be placed with licensed online operators in Australia. Nonetheless, other Australian research has found quite widespread placement of in-play bets in venues, by phone and with unlicensed operators [4], albeit far less than in jurisdictions where their online provision is legal [39, 77]. In-play betting, including cash-out options, facilitates faster and more intensive betting sessions where bettors can rapidly re-stake wins or chase losses on an extended array of continuous betting opportunities [18, 31]. These harmful behaviours are reflected in rates of gambling problems amongst in-play bettors that are 3–4 times higher than amongst non-in-play bettors [4, 45].

The relatively low use of in-play betting in Australia, as found in the current sample, demonstrates that regulation can be targeted to help constrain the growth of problems and harm associated with online gambling. Based on this and previous research, further regulation could contribute to harm reduction goals by curtailing the provision of exotic bets, including multi-bets [32, 39, 41], and reducing advertising and inducements for online gambling [8, 44, 71–73]. For example, banning the sponsorship of sport by gambling companies and prohibiting direct marketing for online gambling would greatly reduce the current proliferation of advertising and inducements. Regulation to ensure that bettors can withdraw funds from their betting accounts easily and quickly, and not cancel withdrawals, would also help customers to better control their online gambling expenditure and limit the consequent financial harm.

In contrast to regulatory and industry objectives to minimise gambling harm, industry changes over the last decade were reported to undermine self-regulatory efforts and exacerbate harmful behaviours amongst online gamblers struggling to maintain or regain control over their gambling. Conversely, non-treatment-seekers reported limited detrimental effects. The study’s findings therefore suggest that industry changes that have made online gambling easier, faster, and heavily incentivised, and the provision of an increasing array of exotic bets with poorer odds, unduly affect addicted and harmed individuals who are also the most profitable customers due to their elevated gambling losses [78–80]. Given that people with a gambling problem report experiencing impaired control over their gambling and limited use of harm minimisation features, consumer protection needs to extend beyond self-regulatory tools, to regulate for safer online gambling products and industry practices.

### Limitations

Exploring all types of online gambling was constrained by the prohibition on the online provision of casino-style games, slot machines and in-play betting in Australia, although Australians can easily access unlicensed operators who provide them. Generalisability of the findings is limited due to the small, purposive interview samples that were self-selecting and predominantly male, and data saturation may not have been achieved. Larger samples may provide more certainty of data saturation, and identify additional themes, perspectives, experiences and comments. The mean age of the two groups differed, leading to a potential age bias between the sub-samples. The non-treatment-seeking group was considerably older, due to the inclusion criteria for these participants to have gambled online for around 10 years. The use of different interviewers for the two sub-samples may have impacted the results. The results may also be subject to recall and social desirability biases. However, drawing on the lived experience of participants has enabled richer insights than can be obtained in quantitative studies and identified potentially harmful changes in online gambling that are worthy of further examination.

### Further research

Whether online gambling has become more harmful remains an open but important question. Measuring changes in gambling harm over time would enable a better understanding of how features of online gambling may affect negative consequences amongst different gambler risk groups. Further, early studies suggested that online gambling does not elevate gambling problems, instead concluding that gambling involvement, rather than online gambling per se, explains higher rates of gambling problems amongst online gamblers [35, 81–86]. However, more recent population research has found that engaging in online gambling is uniquely associated with higher gambling severity after controlling for the number of gambling forms and key demographics [86]. Whether these contrasting results are due to more recent changes in online gambling is unknown but warrants further research to inform policy and regulation that target particularly harmful features and improve consumer protection.

## Conclusion

Key changes in the provision of online gambling over the past decade have included its increased ease and speed, the continued proliferation of advertisements and inducements, and the introduction of numerous innovated bet types. Higher-risk online gamblers disproportionately reported negative effects from these changes, since they fostered increased gambling, impulsive gambling, persistence, and loss-chasing. Recent changes to online gambling that exacerbate harmful gambling behaviours amongst vulnerable online gamblers are counter to stated policy and practice objectives to minimise gambling harm, while current harm minimisation tools have limited uptake and effect. Further consideration is needed to ensure gambling policy, industry practices and public health measures more effectively reduce gambling harm in contemporary settings. In particular, the proliferation of inducements and the poor pricing of complex bets such as multi-bets, and their outsized attraction to players with problems, should be a key area of focus. These incentivised bets target problem players with poor odds, whereas successful gamblers are banned from play by online betting providers. This combination is clearly in opposition to customers’ reasonable expectations for fair-play in betting.

## Data Availability

The data that support the findings of this study are available from Gambling Research Australia, but restrictions apply to the availability of these data, which were used under license for the current study, and so are not publicly available. Data are however available from the authors upon reasonable request and with permission of Gambling Research Australia.
